# Nutritional, Gastrointestinal and Endo-Metabolic Challenges in the Management of Children with Spinal Muscular Atrophy Type 1

**DOI:** 10.3390/nu13072400

**Published:** 2021-07-13

**Authors:** Antonio Corsello, Lorenzo Scatigno, Martina Chiara Pascuzzi, Valeria Calcaterra, Dario Dilillo, Sara Vizzuso, Gloria Pelizzo, Elena Zoia, Anna Mandelli, Annalisa Govoni, Alessandra Bosetti, Ruggiero Francavilla, Flavia Indrio, Valentina Fabiano, Gian Vincenzo Zuccotti, Elvira Verduci

**Affiliations:** 1 Department of Pediatrics, Vittore Buzzi Children’s Hospital, University of Milan, 20154 Milan, Italy; antonio.corsello@unimi.it (A.C.); lorenzo.scatigno@unimi.it (L.S.); martina.pascuzzi@unimi.it (M.C.P.); valeria.calcaterra@unipv.it (V.C.); dario.dilillo@asst-fbf-sacco.it (D.D.); sara.vizzuso@unimi.it (S.V.); annalisagovoni95@gmail.com (A.G.); alessandra.bosetti@asst-fbf-sacco.it (A.B.); valentina.fabiano@unimi.it (V.F.); gianvincenzo.zuccotti@unimi.it (G.V.Z.); elvira.verduci@unimi.it (E.V.); 2 Pediatric and Adolescent Unit, Department of Internal Medicine, University of Pavia, 27100 Pavia, Italy; 3 Pediatric Surgery Unit, Vittore Buzzi Children’s Hospital, University of Milan, 20154 Milan, Italy; gloria.pelizzo@unimi.it; 4 Department of Biomedical and Clinical Science “L. Sacco”, University of Milan, 20157 Milan, Italy; 5 Division of Pediatric Anesthesia and Intensive Care Unit, Department of Pediatrics, Children’s Hospital Vittore Buzzi, 20154 Milan, Italy; elena.zoia@asst-fbf-sacco.it (E.Z.); anna.mandelli@asst-fbf-sacco.it (A.M.); 6 Department of Pediatrics, “Giovanni XXIII” Children Hospital, “Aldo Moro” University of Bari, 70126 Bari, Italy; 7 Dipartimento di Scienze Mediche e Chirurgiche, University of Foggia, 71122 Foggia, Italy; 8 Pediatric Clinical Research Center Fondazione Romeo ed Enrica Invernizzi, University of Milan, 20157 Milan, Italy; 9 Department of Health Sciences, University of Milan, 20146 Milan, Italy

**Keywords:** SMA, spinal muscular atrophy type 1, SMN1, nutritional management, enteral nutrition, pediatric gastroenterology, dysphagia, neurological disability, endocrine disorders, precocious pubarche

## Abstract

The management of patients with spinal muscular atrophy type 1 (SMA1) is constantly evolving. In just a few decades, the medical approach has switched from an exclusively palliative therapy to a targeted therapy, transforming the natural history of the disease, improving survival time and quality of life and creating new challenges and goals. Many nutritional problems, gastrointestinal disorders and metabolic and endocrine alterations are commonly identified in patients affected by SMA1 during childhood and adolescence. For this reason, a proper pediatric multidisciplinary approach is then required in the clinical care of these patients, with a specific focus on the prevention of most common complications. The purpose of this narrative review is to provide the clinician with a practical and usable tool about SMA1 patients care, through a comprehensive insight into the nutritional, gastroenterological, metabolic and endocrine management of SMA1. Considering the possible horizons opened thanks to new therapeutic frontiers, a nutritional and endo-metabolic surveillance is a crucial element to be considered for a proper clinical care of these patients.

## 1. Introduction

Spinal muscular atrophy type 1 (SMA1), also known as Werdnig–Hoffman disease, is an autosomal recessive neuromuscular disease caused by a homozygous mutation or deletion in the survival motor neuron 1 gene (SMN1), which is located on chromosome 5 together with another gene involved in the production of SMN protein, named SMN2 [1,2]. It has an estimated incidence of 1 in 7000 newborns, with a carrier frequency of 1:45 among the Caucasian population [3]. Type 1 is the most severe subtype of SMA, with a symptoms onset that generally occurs before the age of 6 months and with the lowest survival rate in the first years of life. Milder and less common clinical types of SMA have been described (SMA2, SMA3), with a late onset of disease and increased expectancy of life if compared to SMA1 patients [3,4].

Hypotonia is generally identified by caregivers as the first symptom in about 60% of cases of SMA1, with a 90% of onset occurring within the sixth month of life [5]. Thanks to recent methods of diagnosis and to a wider availability of genetic tests, the mean interval between the onset of clinical signs and the diagnosis made by SMN1 molecular analysis is now less than 6 months [5].

Despite recent progress in research and therapy, indeed, SMA1 mortality rate at two years of life still reaches 50% [6,7]. The survival rate is significantly different depending on whether the patient presents a severe form of SMA1 or an intermediate one [8]. Main causes of death described among these patients are respiratory distress, bulbar disorders and respiratory infections. In patients with intermediate forms of SMA1, instead, only 26% died during follow-up, while only half of the remaining patients (57%) needed a tracheostomy [9].

Many gastrointestinal (GI) related disorders, such as dysphagia and aspiration pneumonia, conditions like underweight and overweight or metabolic and endocrine disorders are commonly identified in patients affected by SMA1 [2]. For this reason, a proper multidisciplinary approach is required in the clinical care of these patients, with a specific focus on the prevention of most common complications.

In recent years, new therapies have been approved, and many drugs have shown unpredictable efficacy; thanks to their introduction, the management of young patients has been enriched with approaches that can modify the natural history of the disease, improving the quality of life and extending life expectancy.

One of the main new therapies is represented by nusinersen (Spinraza^®^), an antisense oligonucleotide designed to increase the expression of the SMN2 protein. It was approved by the Food and Drug Administration (FDA) in December 2016 and by the European Medicines Agency (EMA) in June 2017 [10]. A study involving 121 infants (mean age 7 months) with SMA1 showed that nusinersen is effective in improving muscular movement when compared with placebo. After one year of treatment, 51% of infants who received nusinersen (37 out of 73) had progress in developing head control, rolling, sitting, crawling, standing and walking, while no similar progress was observed in infants who received placebo. Additionally, most infants treated with nusinersen survived longer and required assisted breathing later than those who received placebo [10,11]. Nusinersen recommended schedule of treatment consists of four total loading doses, with the first three loading doses administered at 14-day intervals and the final dose that should be administered 30 days after the third dose. A maintenance dose is then administered once every 4 months [12].

In 2019 the FDA and in 2020 the EMA approved onasemnogene abeparvovec (Zolgensma^®^) for the treatment of SMA1 [13]. Onasemnogene abeparvovec is an adeno-associated viral vector-based gene therapy designed to deliver a functional copy of the SMN1 gene to the motor neurons through a single intravenous infusion [14]. The majority of SMA1 patients treated with therapeutically dosed onasemnogene abeparvovec reached the sitting position without assistance; those who were treated earlier reached this milestone much more quickly [15]. A further study has shown the rapid efficacy of gene therapy, capable of maintaining its effects over a long period; 24 months after the start of the study, all patients remain alive and are free from artificial ventilation [16].

Risdiplam (Evrysdi^®^), approved by FDA in august 2020 and by EMA at the beginning of 2021, is a small molecule that modulates the SMN2 gene splicing, subsequently increasing the levels of full-length SMN mRNA and related protein [17]. Regarding the efficacy of this drug in a study carried out on children with SMA1 after 12 months of treatment with risdiplam, the overall survival rate was 90.5%, and no children needed permanent ventilation or had lost breathing and swallowing ability [18]. Even after 23 or more months of treatment, 81% of patients were alive without permanent ventilation [19,20].

Furthermore, with the advent of new therapies, the application of a sensible neonatal screening will be useful and crucial tool in the next future. United States, Germany, Poland, Serbia and Australia have already decided to implement neonatal screening programs for SMA [21,22]. Some pilot programs have also been activated in other countries, such as Italy, Spain and Belgium. When screening programs are fully operational, it will be possible to identify earlier affected children, allowing to take care of patients before the onset of symptoms and achieving better clinical results.

Evidence of the crucial importance of neonatal screening in patients with SMA1 comes from a study conducted in 2021 in Germany by Katharina Vill et al. [23]. This study analyzed the gene involved in the pathogenesis of SMA: in case of a test positivity, the families have been subsequently contacted for a diagnostic confirmation at the reference center. From the analysis of the data, it emerged that through an early diagnosis it was possible to start therapy before the development of symptoms, thus allowing a normal neurodevelopment in all children enrolled. The high sensitivity and specificity of the screening method also emerged, and no false positive nor false negative have been found in the cohort observed.

Further confirmation can be found in a study by Darryl C De Vivo et al. [24], who evaluated the survival rate or need for mechanical ventilation in patients treated with nusinersen after an early genetic diagnosis. Patients treated early had a significantly different disease-free survival time compared to untreated patients, with subsequent a neurodevelopment comparable to healthy controls and no signs of regression in the follow-up period.

However, despite the benefits brought by new therapies and neonatal screening, it is important to underline that not all patients with SMA1 can be currently treated at this moment, and screening programs are still in their beginning stages in many countries. 

For this reason, there will probably be a moment of transition in the next few years where we will be called to face with situations of extremely different spectrum of disease, related to the eventual introduction of pharmacotherapy and the timing of diagnosis. In this context, it will be very important to take charge of patients in a multidisciplinary approach, in to allow correct patient management regardless of the degree of severity.

It will be important to define whether the child with SMA in therapy will have a normal growth or if it will be followed over time to guarantee a normal development. Furthermore, because the advent of new therapies, which has created a type of patient with different life expectations, the gastroenterological and endocrine aspects are key issues to be monitored, trying to understand particular needs of these patients.

**Figure 1:**
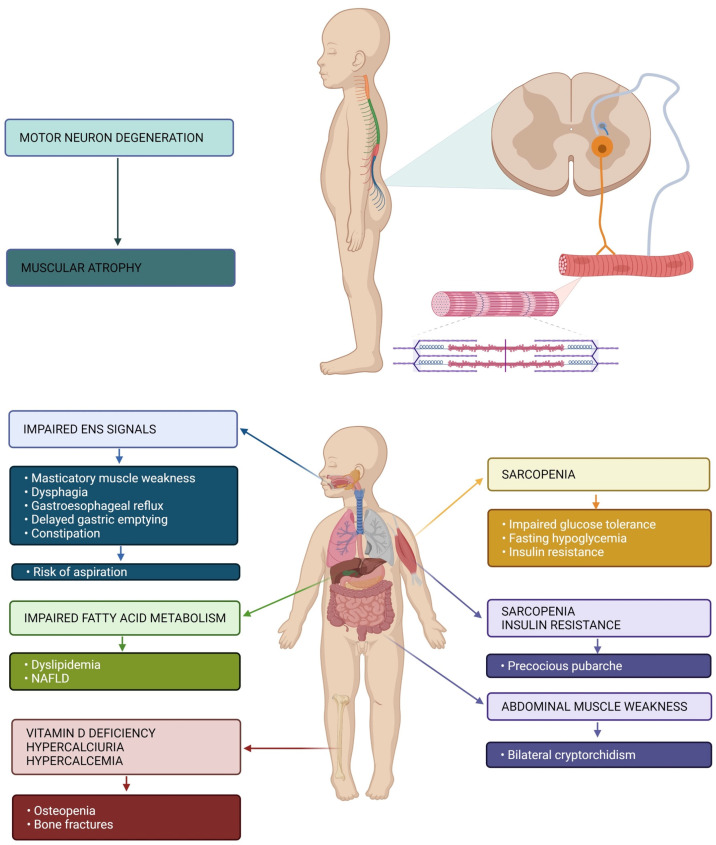
Pathophysiology of muscular atrophy and GI, metabolic and endocrine symptoms of patients with SMA1. Impaired enteric nervous system (ENS) signaling represents the main cause of GI manifestations (weakness of masticatory muscles, dysphagia, gastroesophageal reflux, delayed gastric emptying and constipation). These may be responsible for the risk of aspiration. Impaired fatty acid metabolism may cause dyslipidemia and non-alcoholic fatty liver disease (NAFLD). Altered bone metabolism may result in osteopenia and increased risk of bone fractures. Sarcopenia is responsible for metabolic manifestations (impaired glucose tolerance, fasting hypoglycemia and insulin resistance) and endocrine (precocious pubarche). Weakness of the abdominal muscles may also be responsible for bilateral cryptorchidism.

Figure 1 sums up pathophysiology and main symptoms of SMA1 patients.

## 2. Methods

The approach of this narrative review was to explore the literature and highlight the insights from articles that, in our opinion, made an important contribution in the management of SMA1. A search of PubMed database using various keywords was performed, evaluating papers published up to May 2021 in each author’s field of expertise.

Search terms utilized have been: “SMA1”, “nutrition SMA”, “metabolism calculation in SMA”, “devices for enteral nutrition”, “formulas for enteral nutrition”, “gastrointestinal symptoms SMA”, “metabolic complications SMA” and “novel therapies SMA”. Considering the high number of articles found, only most relevant and cited articles in English published were then collected, in order to better identify different aspects of SMA1 pathophysiology and all principal recommendations published in recent years.

After an initial drafting of different section of this paper, we proceeded to merge the contents, exposing our work to the criticism of various authors. The resulting standardized version was then further improved thanks to the expertise of specific specialist in each field. 

## 3. Nutritional Aspects: Critical Issues and Possible Solutions

### 3.1. Undernutrition and Overnutrition: A Common Problem

Many studies have reported various forms of undernutrition and overnutrition in SMA patients, suggesting a multifactorial impairment that can produce different nutritional phenotypes [25,26]. For this reason, energy needs should be properly evaluated in each patient.

The risk of under or over-estimation of the energy intake could be linked both to the reduction of free fat mass (FFM) and to an excessive expenditure of fat mass (FM). The loss of lean body mass can also compromise the respiratory strength of the already weak muscles [27].

Sometimes a wrong anthropometric evaluation can occur due to the loss of lean mass, especially inadequate growth charts are used. Without a careful evaluation, the loss of muscle can mistakenly suggest a condition of malnutrition. Therefore, anthropometric parameters must be reported on the Center for Diseases Control (CDC) growth charts (as there are no specific ones for SMA1), and the achievement of the 3rd percentile can be considered as an acceptable target [28].

A recent cross-sectional study conducted by Baranello et al. evaluated the association between different body composition parameters and SMA motor function assessment, trying to identify new potential biomarkers of disease severity [29]. The study enrolled 88 children with SMA1 and 2, demonstrating that a physiological body composition is positively associated with better motor abilities; the researchers found that in children with SMA the FM is higher than in healthy controls. Although it has not been proven yet, the FM could have a negative impact on the motor ability of patients with SMA, as suggested by Sproule and colleagues [30]. Another interesting association found is between reduced FFM and a reduction in motor skills, suggesting how the measurement of body composition can be a valid marker of SMA severity.

In addition to this, in SMA1 patients, a severe risk of malnutrition caused by masticatory muscle weakness, dysphagia and breathing problems has to be considered, which could lead to a reduction of the total energy intake. Furthermore, an increased work of breathing could increase energy requirements and expenditure [2].

All these aspects show how the nutritional support in SMA1 is a fundamental component of multidisciplinary care. It is crucial to estimate energy needs in a correct and accurate way, in order to ensure an adequate nutritional support for these patients [31].

### 3.2. Ventilated Patients and Non-Ventilated Patients: What Are the Differences?

Without any treatment, 90% of patients with SMA1 die before 12 months of life, and 100% die by the second year [32]. Furthermore, in the management of the patient with SMA1, artificial ventilation via tracheostomy or non-invasive ventilation (NIV) are frequently needed [33]. The tracheostomy allows to increase the survival rate and average age expectancy; indeed, some children with SMA1 have survived thanks to tracheostomy up to 20 years of age [32]. However, tracheostomy does not allow the development of speech and forces the child to depend on the respirator for the rest of his life. In addition to this, complications related to tracheostomy frequently become themselves the cause of patient’s death. NIV helps the patient’s respiratory mechanics and improves ventilation, and unlike the tracheostomy, it allows the development of verbal skills and improves the quality of life [34].

Although it is undeniable that ventilation allows a significant increase in life expectancy, it must be said that the use of invasive artificial ventilation always has to be carefully evaluated. Indeed, it has been shown that diaphragmatic inactivity determines atrophy of the muscle fibers, and even a few hours of complete rest of the diaphragm can lead to the loss of half or more of the muscle fibers [35]. For this reason, it becomes very important to support the patient’s respiratory muscles without completely replacing them; Tobin et al. suggest a careful calibration of parameters of the ventilation machine, in order to avoid an excessive muscles strain or, on the other hand, their complete disuse [35]. 

Regardless of the type of ventilation, it is then important to highlight that when the respiratory mechanics no longer depends exclusively on the respiratory muscles, total energy expenditure changes significantly. Bertoli et al. have shown that the thoracic-abdominal movements of SMA patients during spontaneous breathing, compared to the reduced ones of mechanical ventilation assisted patients, can significantly increase the resting energy expenditure (REE). Indeed, in this study, authors observed that the use of mechanical ventilation has led to a reduction in REE that reaches the 50% [25].

At the basis of this observation, there seems to be an alteration of the respiratory mechanics. Indeed, in patients with SMA1, a paradoxical breath can be found; this breath turns out to be less effective but more expensive on the energetical side; however, the alteration of respiratory kinetics disappears as soon as artificial ventilation is started; this could be the reason to the significant REE reduction observed [6,36].

**Figure 2:**
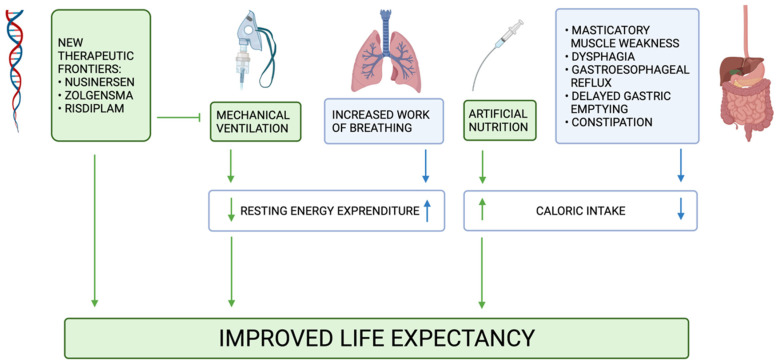
Mechanical ventilation and artificial nutrition, by decreasing the REE and increasing the energy intake respectively, have improved the life expectancy of patients with SMA. The common element of new therapeutic frontiers is to delay the use of mechanical ventilation.

This aspect must therefore be kept in mind in order to avoid malnutrition in unventilated children or, on the other hand, without supplying to a ventilated child a higher than necessary energy intake. Figure 2 simplifies how mechanical ventilation, together with artificial nutrition and GI symptoms, can modify REE and energy intake in SMA1.

### 3.3. Predictive Energy and Fat Mass Equations

The indirect calorimetry represents the gold standard for the determination of REE, but it is not always available in the hospital setting [37]. Therefore, in order to get an estimate of the amount of energy required by the body to maintain physiological functions, predictive equations are needed in order to determine the REE in children, such as the Schoefield formula [38].

The International Standards of Care for SMA recommend an estimated amount of 9–11 kcal per cm [25]. An accurate estimation of REE in SMA patients must consider demographic variables (age, sex), anthropometric measurements (body weight, supine length, tibia length, body mass index), respiratory function (spontaneous, non-invasive ventilation, invasive ventilation / tracheostomy), type of nutrition (oral, nasogastric tube, gastrostomy) and a possible therapy with nusinersen.

Bertoli et al. analyzed some of these characteristics and their impact on the REE in SMA1 patients [25]. As explained above, this study showed that the use of assisted ventilation is what principally influences REE. For this reason, different formulas with different coefficients have to be then considered by the clinician when evaluating energy needs of spontaneously or mechanically ventilated patients [39].

Finally, a new equation for FM prediction in SMA1 patients has been developed by Foppiani et al., considering that weight and body mass index are misleading biomarkers in these patients [29,40]. This study, which included 152 patients with SMA1, provides to the clinicians a new useful tool in order to precisely define body composition among these children.

### 3.4. Enteral Nutrition

As already described, the progression of disease can cause breathing difficulties, including ineffective cough reflex; this alteration, together with a worsening of swallowing symptoms, dysphagia and hypotonia, increases the risk of inhalation and aspiration of food material. Therefore, in order to ensure a safe breathing and proper nutrition, an artificial nutrition early becomes fundamental in the management of children with SMA1 [41]. 

Another interesting role of artificial nutrition in SMA1 has been explained by MC Ørngreen et al. [42]. This study shows how in SMA1, as in all neurodegenerative diseases, a greater tendency to develop hypoglycemia during the fasting phases can be observed; the reason of this phenomenon seems to be to the lack of muscle mass as an important supplier of substrates for gluconeogenesis; therefore, due to the extremely reduced muscle mass, gluconeogenesis is less effective in these patients [26]. For this reason, an important role of artificial nutrition is to prevent prolonged fasting and, subsequently, hypoglycemia.

A crucial aspect in SMA1 management is defining the most correct time to introduce enteral nutrition (EN) by tube [43]. A contribution that can be useful in this delicate decision comes from a study conducted by Wadman et al., which found that maximum mouth opening correlates with an increased risk of dysphagia and choking [44]. Therefore, mouth opening assessment represents a simple technique which can help to identify patients at higher risk of aspiration and feeding difficulties.

#### 3.4.1. Nasogastric and Nasojejunal Tube vs. Percutaneous Endoscopic Gastrostomy (PEG) and Percutaneous Endoscopic Transgastric Jejunostomy (PEG-j)

Firstly, until an adequate weight for safely performing surgery is achieved, the placement of a nasogastric tube or a nasojejunal tube may be chosen. The choice between these two options mainly depends on the presence or absence of gastroesophageal reflux (GER); in fact, in presence of reflux, the nasogastric tube could still cause inhalation of food material and subsequent complications [45]. On the other hand, the nasojejunal tube guarantees a greater protection of the airways despite having important limits in terms of usable mixtures and administration schedules: the osmolarity of the mixture and the speed of administration through the nasojejunal tube, which must necessarily be reduced without the reservoir function of stomach. 

Enteral tubes should be considered a temporary solution, and as soon as possible, a definitive stoma should be placed. A definitive stoma has numerous advantages, significantly improving the quality of life by freeing the child from a permanent foreign body in the oral cavity and allowing a more effective nutrition. This can be observed through an improvement of anthropometric parameters in children to whom the stoma is placed: indeed, a doubling of the percentiles in length and weight can be observed after surgery [46].

Furthermore, even if there is no unanimous consensus, many experts recommend performing “Nissen fundoplication” concurrently with the gastrostomy placement. Indeed, if a “Nissen fundoplication” is performed simultaneously with the stoma placement, a clear reduction of GER can be obtained, significantly reducing the risk of pneumonia and the mortality rate. A practical demonstration of this evidence comes from the Durkin et al. study, where “fundoplication” surgery reduced hospitalization rates by more than 50% [46].

However, each case should be evaluated individually considering the high perioperative risk, prognosis, and life expectancy. Furthermore, even the most aggressive approach to GER in SMA1 does not guarantee the prevention of complications [47]. 

#### 3.4.2. Administration Schedules

The management of EN in the child with SMA1 is an extremely complex issue; its management must be placed in the hands of an expert team in artificial nutrition. Each administration scheme must in fact be calibrated on the patient, according to his nutritional needs, the type of formula used, and the type of device used, paying particular attention to the needs of the family and the patient. Furthermore, in order to be effective, EN must also be well tolerated; it is essential not only to correctly set up nutrition but also to guarantee a regular follow-up [48].

EN can be continuous or discontinuous; the first one is the most easily tolerated, and it can also be carried out using post-jejunal probes; however, it represents an important limitation as it forces the patient to be continuously attached to an infusion pump. Discontinuous administration, on the other hand, is the one that comes closest to the human physiology, as it allows the infusion of larger volumes in shorter periods of time [49]. It therefore allows the child to have free time from the tube to be able to play and explore the environment that surrounds him. However, discontinuous EN has some limitations: it is more difficult to be tolerated by the patients and, in presence of GER, it exposes to a higher risk of inhalation. In addition to this, the administration of boluses exclusively during the daytime also exposes to an increased risk of hypoglycemia [27,50].

An excellent compromise, if tolerated, is the combination of the two techniques; in fact, discontinuous administration during the daytime improves the patient’s quality of life, while the continuous nocturnal administration prevents the risk of hypoglycemia and reduces the risk of inhalation given by the supine position [51].

#### 3.4.3. Enteral Formulas

The choice of formulas for enteral nutrition (EN) depends on the age of the child, his metabolic state and the GI function [52]. For the infusion of formulas through tube, it is suggested to use flavor-free ones. Standard formulas are derived from milk products and they’re lactose and gluten-free. The standard polymer formulas provide 1.0–1.5 kcal/mL [53]; they are lactose and gluten-free and can be enriched with insoluble fiber. The semi-elementary formulas are based on peptides, and they have been designed for patients suffering from malabsorption or digestion problems. Elementary formulas are made of amino acids, and they can be used in case of malabsorption problems [54].

In the case of SMA1, four studies reported the diet with an elementary formula as a possible strategy for managing nutritional-gastroenterological problems [45,55,56,57]. Parents have described several benefits, including a reduction of heavy sweating and flushing episodes, lung secretions, cough and abdominal distension [31]. Moreover, it seems that the use of elementary formulas with a low lipid content can be better tolerated by SMA1 children. In particular, all patients who have an abnormal gastric emptying can benefit from low-fat diet, which can help to improve motility and reflux [58]. Unfortunately, additional data are needed to confirm these benefits. In addition to a correct nutritional intake, another essential element in the management of disease is to provide an adequate hydration and a correct electrolyte balance [2].

Generally, daily fluid requirements are 115–135 mL per kg body weight for a normal maintenance, but this request could change in presence of fever or dehydration.

## 4. Gastrointestinal Aspects and Clinical Challenges

### 4.1. Symptoms, Mechanisms and Pathophysiology

GI symptoms observed in SMA1 patients mainly include constipation, GER, delayed gastric emptying, dysphagia and vomiting [2]. All these symptoms could lead to a higher risk of aspiration and subsequent pneumonia, which is still the first cause of death among these patients [59]. Masticatory muscle weakness, dysphagia and respiratory problems could result in a reduced energy intake and subsequent malnutrition (Figure 2). The contracture of the masseter muscle is another symptom that frequently occurs within one year of age among these patients and that brings a more difficult oral feeding [2]. Other GI disorders that can be observed in these patients are intolerance to bolus feeding and poor motility. 

All these symptoms seem to be caused not only by a defect of the SMN in the central nervous system, but also a pathogenetic role performed by the enteric nervous systems (ENS) has been proven. Studies in mice have shown that SMN deficiency can result in disruption of ENS-mediated signaling to the smooth muscle of the colon but without causing loss of enteric neurons [60]. 

In mice models affected by SMA that presented GI symptoms, an electrical stimulation of distal segments of the colon generated a greater contractile response in SMN deficient tissues, indicating an absent circuit disinhibition [61]. In addition to this, animal experiments conducted in mice with SMN gene mutations also showed an important reduction of bowel villi. A significant increase in macrophage infiltration into the small intestine has also been found [62]. An increased intestinal permeability has been demonstrated in SMA mice, resulting in microbial invasion into the circulatory system. Systemic inflammation in SMA is a pathological mechanism which influences phenotypic severity, and this should also be considered in individualized dietary approaches and drug administration [63]. Moreover, significant microscopic alterations were found in the GI tract of mice with SMA type 1-like phenotype, including a decrease in vascular density. Vascular defects have also been reported in severe cases of SMA1, and it has therefore been suggested that GI disorders could be related to vascular abnormalities [64,65].

#### 4.1.1. Upper GI

Swallowing is a complex physiological action which is regulated by both central and peripheral stimuli, with 6 different cranial nerves and more than 30 muscles involved; it can be generally divided into oropharyngeal and esophageal phases [66]. Since about the sixth month of life, the beginning of the oropharyngeal phase, corresponding to the preparation and the propulsion of the bolus, becomes under voluntary control, while the interruption of respiratory function and the esophageal phase are under involuntary control [67]. Any problem or difficulty in any of these components can lead to dysphagia [66]. 

Multiple and incomplete swallows, poor intraoral bolus control, tongue dysmotility and lips muscular weakness are just some of the elements that could lead to oral dysphagia, generally for both solids and liquids. Ensuring a safe swallowing is then a priority aspect in these patients, in order to avoid aspiration and pulmonary infections. Neuromuscular Disease Swallowing Status Scale (NdSSS) has been used as a useful clinical tool to describe swallowing status in SMA patients, in order to evaluate the progressive swallowing dysfunction and to have a quick overview on the severity of disease and its possible management [68,69]. 

Patients with SMA1 who are not able to sit, also called “non-sitters”, should be evaluated with a videofluoroscopic swallowing study (VFSS) at the time of diagnosis if growth failure has occurred and above all when an evidence of dysphagia appears. With a normal initial test, a close monitoring is mandatory in order to detect possible early signs of feeding difficulties. A failed VFSS is an indication for a short-term placement of a nasogastric tube until long-term gastrostomy tube can be positioned, for primary prevention of GER and its complications [2]. A gastro-jejunal bypass tube should be considered in suspecting intolerance of gastric feeding with evidence of post-feeding abdominal pain [70]. SMA patients with partial mobility despite weakness, also called “sitters”, should have a periodic evaluation of their swallowing safety. Less aggressive management with prokinetic and acid-reducing therapies may be used in this population without the need for surgery. Treatment may change with advancing age, and a reevaluation should be performed [2].

Spontaneous choking crises have also been reported, and when they occurred during meals, they caused severe swallowing difficulties [71]. These episodes lasted several minutes, causing a choking sensation and triggering coughing. The origin of these manifestations may result from episodic autonomic dysfunction, as no significant esophageal manometry abnormalities have been reported in literature.

GER has been shown to be associated to a higher risk of worsening of other symptoms, aspiration and mortality in SMA patients. The true incidence is still unknown; however, it has been reported in almost the entire population of some studies [46]. Symptoms that could hide a possible GER are frequent regurgitation, refusal of food, unexplained vomit and persistent crying. In order to avoid a possible subsequent malnutrition and other complications, possible formulas or food that increase the risk of GER should be avoided. 

Medical management of GER in patients with SMA may include simple strategies such as a change in solid and liquid consistency as needed for the individual patient or the use of thickened liquids positioning during meals in order to protect airways. Regarding possible medical treatments of GER, acid neutralizers (e.g., magnesium carbonate) should be used in severe cases with higher risk of complication, but possibly in the short therm. Given the high prevalence of GER and the difficulty to perform invasive investigations in this group of children, a trial of proton pump inhibitors (PPIs) with careful clinical follow-up is considered acceptable [72].

Therefore, the main purpose of therapy and management in these patients is then to reduce the risk of aspiration, together with guaranteeing the best possible growth through a proper nutrition. The aim of the clinical assessment should be a physical examination of possible gastroenterological manifestations, focusing on the management of gastro-esophageal reflux to prevent the risk of aspiration. Acute worsening of signs and symptoms should lead to presentation to emergency care. Attention should be paid to the risk of aspiration during illness, when diffuse muscle weakness is exacerbated, and oral feeding is decreased. Hospitalization may be required in case of acute disease (e.g., aspiration, community-acquired infection, gastroenteritis with dehydration) and planned surgical procedures (e.g., gastrostomy tube placement, “Nissen fundoplication”). Endotracheal intubation should consider several factors including limited neck and mandibular mobility, positioning restrictions and family will [73].

Few studies have evaluated aspiration risk in SMA1. Choi YA et al. reported that 5 of 11 patients developed recurrent aspiration pneumonia and were subsequently switched to tube feeding [74]. Additionally, patients demonstrated significant variation in deterioration of swallowing function before 12 months of age. Gastrostomy tube feeding has significantly improved the probability of survival beyond two years in patients with SMA1 [48]. Novel therapeutic approaches for SMA1 such as nusinersen have demonstrated a significant improvement in the motor function and acquisition of motor milestones, as well as a longer survival [75,76]. 

Treatment with oral risdiplam results in an increased sera expression of functional SMN protein in infants with SMA1 [17]. Furthermore, a multicenter phase 1 study evaluated the safety and efficacy of a gene therapy with onasemnogene abeparvovec, showing a significant clinical response. A more durable functional independent sitting and permanent ventilation-free time was observed. The favorable benefit-risk profile shown in this study supports its use for the treatment of symptomatic patients with genetic or clinical features predictive of SMA1 [77].

In addition to this, recent research has shown that a single-dose gene replacement therapy increases the probability of not requiring nutritional support [74,75]. Further research focusing on the effect of new therapies on swallowing function is certainly needed, and an individualized approach should be recommended to improve care and life expectancy of patients with SMA1.

#### 4.1.2. Lower GI

Chronic constipation has been generally observed, and it seems to be caused by multiple factors, such as a reduced intake of fiber and fluids, a reduced tone and strength of abdominal wall muscles and a GI dysmotility [78]. In selected cases constipation alternating with episodes of fecal incontinence were reported. Studies have reported constipation in 43% of patients, abdominal pain and meteorism in 15% and 14% of patients, respectively [79]. GI imaging is usually normal, excluding mechanical obstruction. Symptoms may result from functional dysmotility of the GI tract, slowed colonic transit, intestinal pseudo-obstruction or dysfunction of the anorectal motor apparatus. Weakness of the abdominal wall muscles may contribute but alone does not explain cases of incontinence, supporting the role of enteric nerve plexus dysfunction [71]. Adequate hydration, proper use of probiotics and motility-promoting medications are recommended to relieve symptoms of constipation [2].

Pelvic floor muscle atrophy, striated muscle weakness and poor smooth muscle contraction, in both contributing to GI symptoms, result in a high rate of urinary incontinence in patients with SMA1. Slow dysmotility may also affect the stomach, resulting in delayed gastric emptying. It may contribute to GER and early satiety, leading to vomiting after meals. This discomfort can cause a decrease in nutrient intake in affected children, increasing the risk of malnutrition. It can also be exacerbated by high-fat foods, which are used to increase calories. Motility studies can be helpful in documenting delayed gastric emptying. It may be necessary to use prokinetics for the treatment [78].

## 5. Metabolic and Endocrine Aspects

### 5.1. Dysregulation of Lipid Metabolism

Children with SMA have various metabolic abnormalities that can further complicate their clinical condition. One of the major metabolic problems is the dysregulation of lipid profile. An increasing number of studies have shown that patients with severe forms of SMA have metabolic abnormalities involving the metabolism of fatty acids [26,80].

Deguise et al. stated that denervation may contribute to a lower metabolic demand by skeletal muscle, modulating the molecular metabolism upon denervation, or through alterations of metabolic pathways, inducing this dysregulation in the fatty acid metabolism [80]. This phenomenon underlines the need for a monitorization of lipids profile through a meticulous screening, which could help to define a better personalized dietary supplementation. Some studies found that fatty acid metabolism can be defective in SMA1 patients, with disorders like dicarboxylic aciduria, high excretion of urinary acylcarnitine, carnitine deficiency in both muscle and serum and deficiency of acyl-CoA dehydrogenase, a β-oxidation’s enzyme [81]. Although abnormal levels of fatty acid metabolites are frequently reported in SMA patients, it has not been identified a specific defect in mitochondrial oxidation due to the absence of metabolic evidence that occurs in presence of a genetic defect of mitochondrial β-oxidation. In fact, in most SMA patients, a normal profile of both acylcarnitine and ketones, which is typically altered in classical disorders of fatty acid transport and mitochondrial oxidation, can be observed. This evidence seems to reflect a correct utilization of fatty acids by the liver under conditions of fasting or stress [26]. However, aciduria of small and medium chained fatty acids has been more strongly associated with SMA1 if compared to milder types of SMA, suggesting that metabolic abnormalities may depend on SMA severity [82,83].

The activity of five key enzymes involved in the regulation of fatty acid metabolism was investigated from the muscle biopsies of five children with SMA (2 with SMA1, 2 with SMA2 and 1 with SMA3) [81]. Enzymes analyzed were long-chain 3-hydroxyacyl-CoA dehydrogenase (LCHAD), short-chain 3-hydroxyacyl-CoA dehydrogenase (SCHAD), 3-ketothiolase and acetoacetyl-CoA thiolase and enoyl-CoA hydratase. Apart from the enoyl-CoA hydratase, the enzymatic activities of LCHAD, SCHAD, 3-ketothiolase and acetoacetyl-CoA thiolase were reduced in all patients [61]. Acylcarnitine levels were also assessed and were found to be high especially in SMA1 patients, mainly for the percentage of esterified carnitine [82,83]. In another study, mild-to-moderate elevated serum acylcarnitine (mostly C5-OH acylcarnitine and C3 propionylcarnitine) have been found in SMA patients with a severe phenotype [84]. Conversely, reduced carnitine and acylcarnitine levels in muscles and an increased urine excretion of acylcarnitine have been described in SMA patients [85].

This has led to the belief that the lipid metabolism dysregulation is somehow linked to the SMN deficiency. Nevertheless, a study showed that the loss of SMN cannot be considered the specific cause of these defects, as 50 SMA patients revealed similar abnormalities about fasting and non-fasting fatty acid profiles in serum and urine samples compared to healthy controls and infants suffering from a denervating condition [84]. Another evidence supporting the hypothesis that metabolic dysfunction reflects the SMA severity is the ratio of dicarboxylic acids/ketones, which was abnormal in all severe SMA children under 10 months, as for patients with known fatty acid oxidation defects, while infants with milder types of SMA of a similar age had ratios that were within the normal limits [81,84]. Furthermore, it seems that the influence on metabolic perturbations may be age-dependent, perhaps due to developmental functions of SMN1 [81]. What has not been clarified yet is what the specific cause of these metabolic disturbances could be.

Defects in the transport of fatty acids and mitochondrial oxidation could contribute to the muscle wasting typical of severe SMA patients. This fact would also explain the increase in FM despite the very low-energy consumption, even if the exact mechanism has not been clarified yet. The activity of carnitine palmitoyltransferase 1 (CPT1), the enzyme that transports long-chain fatty acids into mitochondria, was found to be reduced in muscles of severe SMA1 patients, compared other infants of the same age. Interestingly, the reduction in the activity of CTP1 has been associated with an impairment of the muscle function in neurogenic atrophies [26]. 

Furthermore, a recent study showed reduced levels of GAP43 proteins in the axons of SMA mice models, which is a growth-associated protein post-translationally modified by a long-chain acylcarnitine implicated in neuronal functions. The translation of GAP43 mRNA in these axons appears to be regulated by the SMN. In this way, through the post-translational regulation of the specific motor neuron protein GAP43, dysregulated acylcarnitine could influence SMA phenotypes. However, the lack of consistent results of the metabolites of carnitine and acylcarnitine seems particularly supported by the hypothesis of reduced oxidation in the pathogenesis of SMA [26].

Unfortunately, tests such as urinary organic acids, muscle β-oxidation enzyme function or plasma acylcarnitine and free fatty acid profiling are less used and not usually accessible, so that they are not suggested choices in the screening and identification of SMA patients with potential metabolic abnormalities [80]. However, an underestimation of these metabolic defects could compromise the systemic overview of each patient’s health, and for this reason, a comprehensive screening of SMA patients could be essential for the management of disease.

### 5.2. Dysregulation of Glucose Metabolism

Recent studies have shown events of fasting hypoglycemia even after a short-term fasting (>4 h but <6 h) in patients with SMA1, postulated as an association with impaired gluconeogenesis [86].

A recent study reported that also in SMA2 and SMA3 underweight patients could have fasting hypoglycemia episodes, and the non-ambulatory overweight/obese patients were particularly inclined to develop at least one of the abnormalities in glucose and lipid metabolisms, such as prediabetes, dyslipidemia or hepatic steatosis [87].

The skeletal muscles are an important source of gluconeogenic substrates during fasting, and hypoglycemia should be considered in SMA patients with severe muscle wasting, especially during stressful events, such as surgery and fever. Therefore, it is recommended that patients with recurrent episodes of hypoglycemia are provided with regular carbohydrate and protein intake, including overnight meals [26]. 

Studies in SMA experimental model have revealed metabolic defects characterized by fasting hyperglycemia, glucose intolerance, insulin hypersensitivity and hyperglucagonemia. Loss of insulin-producing β-cells and a corresponding increase in the number of glucagon-producing α-cells may explain the pathogenesis [88]. Based on the observation that metabolic features occurred before the onset of neurologic symptoms, pancreatic phenotype may be a direct consequence of SMN deficiency [89]. 

Although these defects might be independent from the disease onset, their impact on progression should not be underestimated. Various therapeutic strategies such as the administration of insulin-like growth factor 1, neuronal depletion of phosphatase or tensin homolog and an administration of trichostatin A have shown beneficial effects in SMA mice models, with positive effects on the glucose metabolism [90,91]. Thus, although the SMA therapeutic strategies described above were aimed at motor neuron and muscle pathology, it is possible that the positive results were due in part to improvements in glucose metabolism and pancreatic development. 

Impaired glucose tolerance and insulin resistance has been reported in patients with SMA [89]. Brener et al. [92] reported the presence of with a bimodal distribution according to the weight status, with increased IR in the severely underweight patients (types 1 and 2) and in patients with obesity (types 2 and 3). These results support the hypothesis that the skeletal muscle is a major target of insulin action, and severe sarcopenia and sarcopenia with excess adiposity can lead to increased IR hyperglycemia and diabetes [93]. Insulin resistance was found to be associated with IGF-1 levels in insulin resistant SMA patients. IGF-1 overexpression was suggested in the past as a modulator of SMA severity in mice, the finding that insulin resistance is associated with IGF-1 levels raised concerns that insulin resistance may attenuate the IGF-1 myotrophic effect [94]. 

Future development and evaluation of SMA therapies should therefore also evaluate their effect on glucose metabolism and pancreatic abnormalities [89].

### 5.3. Liver Disorders

Studies have shown that liver SMN deficiency could cause dysregulated hepatocyte metabolism, which could predispose cells to fat accumulation. SMA1 and 2 patients are more likely to develop dyslipidemia, and nonalcoholic fatty liver disease (NAFLD) has also been experimentally reproduced in mouse models, since this increased susceptibility has occurred with an incidence of even 37% [80].

Previous preclinical studies had already shown that fatty acid alterations in SMA patients could not be explained by denervation alone, as denervated control patients did not display these defects [80,84]. According to these findings, specific SMA SMN2B/− mice models developed NAFLD before denervation [26]. In SMN2B/− mice models, it has also been observed that peripheral lipolysis, and the consequent increase in circulating lipids, may be due to hyperglucagonemia in response to low blood sugar levels [26]. The hypoglycemia could come from a perturbation in the pancreas–liver axis. In addition, mitochondrial defects probably contribute to a reducing β-oxidation in hepatocytes. Then, the development of NAFLD may depend on lipid metabolic alterations between peripheral lipolysis and lipogenesis and the export or utilization of fatty acids via β -oxidation.

Recently, in two SMA1 patients following the gene replacement therapy, a subacute liver failure occurred [95]. There is the possibility that the gene therapy made the liver condition worse, since these patients are already predisposed to develop dyslipidemia and NAFLD, or even caused the liver injury. However, in order to make some more targeted hypotheses, more research outcomes are needed, both on the lipid status of the liver of SMA patients and on the metabolic alterations that SMN deficiency can affect.

### 5.4. Vitamin Deficiencies and Bone Health

The nervous system has long been known to be particularly sensitive to deficiencies in folate and vitamin B12, both of which are essential for SMN methylation. Hypomethylation could impair SMN activity and have consequences comparable to reduced levels or mutations in SMN. Inadequate folate and vitamin B12 intake could lead to hypomethylation of the protein and consequently could affect the clinical severity of these patients. Given this possibility, it would seem prudent for these patients to ensure that their diet includes the recommended daily requirement of these vitamins [26,96].

SMA1 patients are at risk for micronutrient deficiencies relevant to bone health [97]. Susceptibility to vitamin D deficiency is due not only to poor intake but also to limited sun exposure, limited absorption and drug-nutrient interactions [98]. These patients require lower energy intake than their healthy peers and may have difficulty achieving adequate micronutrient intake. Biochemical assessment of vitamin D status is a key consideration in confirming a deficiency in at-risk patients. Although reduced serum vitamin D levels often do not reflect inadequate consumption, studies have shown a positive association between micronutrient (such as vitamin D, calcium and magnesium) intake and bone mineral density in patients with SMA1 [27,97]. 

As calcium has a direct effect on bone remodeling, there is a stronger correlation between serum calcium levels and bone mineral density. However, serum calcium is not a good measure of calcium status because the body is very efficient at regulating levels within a narrow range. Serum calcium does not reflect calcium intake or bone health [97]. 

Bone mineral density is a long-term measure of calcium and vitamin D intake. A decrease in bone mineral density increases the risk of osteoporosis, scoliosis and fractures [99]. An increased incidence of bone fractures with laboratory evidence of hypercalcemia and hypercalciuria was observed. Fractures tend to affect the lower rather than upper extremities, with a high prevalence of clinically important femur fractures in children with contractures [100]. A severe SMA1 variant has been described with congenital bone fractures and extremely thin ribs without contractures [101]. 

Patients with SMA have been shown to have almost universally osteopenia. In specific, those with SMA1 had significantly lower BMD at all skeletal sites compared to the other milder phenotypes. Despite the high prevalence of low BMD and bone fractures observed at a young age, only a minority of children met the criteria for osteoporosis in children [102]. In addition, recent data suggest that patients with SMA1 may experience an initial increase in bone density of the distal lateral femur between 3 and 10 years of age, followed by a subsequent decline during adolescence [100]. In less severe cases, however, the ability to walk positively affects bone health [31]. 

Bone metabolism monitoring should be initiated at the time of diagnosis with dosing 25 Hydroxy-vitamin D, calcium, phosphorus, parathyroid hormone and assessing bone mineralization with dual-energy x-ray absorptiometry (DEXA). SMA patients, so-called “sitters” and “non-sitters”, should have a periodic evaluation with both and receive adequate calcium and vitamin D intake for bone health. Less aggressive management may be used in “walkers” population, monitoring only 25 Hydroxy-vitamin D and receiving adequate calcium and vitamin D intake if needed [2].

Studies in mouse models with SMA have aimed to determine the functional role of SMN in bone remodeling [103,104]. An osteoporotic bone phenotype, an increased number of activated osteoclasts and a significant decrease in the osteoblast differentiation markers were shown [80]. However, no changes in alkaline phosphatase expression levels were reported compared with wild-type mice. These studies may suggest SMN function in bone remodeling and skeletal pathogenesis in SMA [103]. 

A study by Hensel et al. found a reduced bone and cartilage development in SMA patients [105]. The authors radiologically examined the bone size and bone mineral density (BMD) in SMA patients and in pre-symptomatic SMA mice. They found that bone growth defect is at least partially independent from neuromuscular degeneration. Moreover, the reduction of the number of chondroblasts in the hypertrophic zone of the growth plate has revealed molecular changes related to cell division and cartilage remodeling, suggesting a bone intrinsic defect in SMA.

However, further studies are needed to determine whether low BMD and propensity for fractures are related to immobility and muscle weakness or direct action of SMN on bone turnover. In addition, more work is required to identify effective interventions to delay BMD decline and prevent fractures in children with SMA. In particular, studies that follow these patients after bisphosphonate treatment are needed to better describe their long-term bone health [100]. 

### 5.5. Endocrine Disorders

Precocious pubarche is a common clinical manifestation in forms of SMA with markedly decreased muscle mass. In healthy girls with premature pubarche, an abnormal body composition of increased body fat percentage with decreased muscle mass has been reported [106]; this phenomenon could be accelerated in the sarcopenic SMA population and premature pubarche can already occur within the first year of life [93]. An increased IR without evidence of hyperandrogenism and an increased prevalence of hypertension have been reported in patients with isolated precocious pubarche [92]. Therefore, precocious puberty could also be considered a precursor of an early-onset metabolic syndrome [107]. Additionally, the role of life stress response in the precocious sexual development could be not excluded [108].

Among females, in milder SMA patients, thelarche and menarche appeared at a normal age, and regular menstrual cycles are usually reported [93]. In males, bilateral cryptorchidism with normal penile size, position of urethral orifice and scrotal development have been commonly seen in severe forms of SMA [93]; ultrasonography frequently found testicles in inguinal position. Severe weakness of the abdominal muscles rather than dysfunction of the hypothalamic–pituitary–gonadal axis is responsible for bilateral cryptorchidism in these patients [92].

Considering that severe cases of SMA patients may, in the future, possibly survive till later age, an endocrine assessment during childhood and adolescence should be evaluated twice a year in all patients, in order to detect eventual disorders of pubertal development or other endocrine problems.

## 6. New Therapeutic Frontiers

Thanks to the great progress of recent years, the child with SMA has at his disposal new and extremely effective therapies capable of changing the natural history of the disease; an interesting starting point for future research may therefore be to find new drugs and dietary interventions that could lead, combined with therapy, to further improve the patient’s outcome.

Olesoxime, a small steroid-like molecule, has been proven in mice models to significantly delay neuronal damage and glial activation [109]; this molecule was then studied on a human model showing a good efficacy, with a neurological improvement and stabilization in a higher percentage than the control group [110].

In addition to the new molecules, it is also important to consider drugs already available; in this regard, it is known that Docosahexaenoic acid (DHA) plays an important role in neuronal development and neuroprotection [111]. An interesting starting point for future research will therefore be to evaluate whether, with the same basic treatment and starting clinical conditions, the use of neuroprotective therapies could improve neurological and muscular outcome.

Furthermore a dietary-nutritional approach could play a crucial role in neuroprotection; some studies on animal models have shown that the ketogenic diet can have a neuroprotective effect not only in diseases such as drug-resistant epilepsy, where it is now widely demonstrated but also in diseases such as Parkinson disease, Alzheimer’s disease, trauma or stroke; the mechanisms are not yet well defined; however, it is plausible that neuroprotection derives from increased neuronal energy reserves, which improve the ability of neurons to resist metabolic challenges, also through an antioxidant and anti-inflammatory effect [112]. 

It will be then possible to act by trying to protect neurons and enhancing muscle response. An example of this is represented by Tirasemtiv, a fast skeletal troponin activator that sensitizes the sarcomere to calcium. This drug works by slowing the release of calcium from the muscle cell, increasing the capacity for muscle contraction. Tirasemtiv, indeed, has been proven in mice models to improve exercise tolerance [113]. Therefore, although it is still to be demonstrated, it can be expected that the therapy may also be useful in patients with SMA. It therefore becomes increasingly important to understand which therapies can be used in order to optimize the effect of disease modifying therapies and above all to ensure that the effect can last over time. Bowerman and colleagues suggest that, in the coming years, further studies will be needed to evaluate the effective impact of combinatorial therapy in the management of patients with SMA both in the therapeutic approach and in the prevention of possible neurodegeneration [114].

Finally, interesting data worthy of mention have been provided by a recent study by Yerushalmy-Feler et al. In this work, a cohort of 51 SMA patients was analyzed, mostly severely malnourished [49]. A nutritional intervention was therefore implemented before the beginning of therapy with nusinersen in one group and after the initiation of therapy in the other one. The study found that the anthropometric parameters underwent a greater improvement in the group where the nutritional intervention was carried out before the introduction of the therapy. Although the study was not random controlled and therefore it needs for further confirmation, it seems to suggest that a nutritional approach before the start of therapy improves the patient’s outcome. 

A challenge for the future, therefore, along with the study of new therapies, must also be to evaluate whether the support through nutritional interventions and a multidisciplinary management will further improve the quality of life of patients with SMA and their expectation of life. Table 1 sums up main elements to be evaluated in the management of the patient with SMA1, according to a gastroenterological, nutritional and endocrine point of view.

## 7. Conclusions

The management of patients with SMA is constantly evolving; in just a few decades, the medical approach has passed from an exclusively palliative therapy to a targeted therapy that has transformed the natural history of the disease, improving survival time and quality of life, and creating new challenges and goals for future treatments [115]. Amazing novelties come from the gene therapy; indeed, new learning about the physiopathology of SMA1 reached a development that was unthinkable a while back for patients with SMA1. This will lead us to face new possibilities about the advances in growth and development of these children and about what will be the best way to guarantee them a proper nutrition, knowing their nutritional needs. 

Metabolic and endocrine aspects must be also considered in order to better define their needs and to ameliorate their life expectancy. These and countless other questions will strike us in the coming years; the role of medicine and research will therefore be able to anticipate them in order to guarantee patients the best chances of survival. Nutritional and endo-metabolic surveillance is crucial to an individualized therapeutic strategy for this vulnerable population. 

## Figures and Tables

**Table 1:** A proposed multidisciplinary approach in the management of patients with SMA1.

Gastroenterological management	- Swallowing study - Gut’s radiologyc assay - PH-impedance - Esophagogastroduodenoscopy - Esophageal and duodenal manometry - Abdomen X-ray - Liver ultrasound (NAFLD screening)
Nutritional management	- Anthro-plicometric measurements - Bioimpendeziometry and indirect calorimetry - Estimated dietary intakes - Complete blood count and nutritional assessment - Targeted dietay supplementation - Planning and activation of therapeutic plans for the supply of nutrition products and devices
Metabolic and endocrine management	- Thyroid function (TSH, FT3, FT4) - Calcium and phosphate homeostasis (serum and urinary calcium, phosphorus, PTH, 25-OH vitamin D) - Prolactin - Adrenal function (ACTH, cortisol, DHEA, DHEAS, 17OHP) - Gonadal function (FSH, LH, 17β-estradiol, testosterone/free-testosterone,) - Glucose-insulin metabolism (glycaemia, insulin, c-peptide, HbA1c) - IGF-1 - Lipid metabolism (triglycerides, HDL, LDL, total cholesterol) - Left carpal X-ray for bone age assessment - Dual-energy X-ray absorptiometry (DXA)

## Data Availability

No new data were created or analyzed in this study. Data sharing is not applicable to this article.
